# Compression Garments Reduce Soft Tissue Vibrations and Muscle Activations during Drop Jumps: An Accelerometry Evaluation

**DOI:** 10.3390/s21165644

**Published:** 2021-08-21

**Authors:** Liqin Deng, Yang Yang, Chenhao Yang, Ying Fang, Xini Zhang, Li Liu, Weijie Fu

**Affiliations:** 1 Key Laboratory of Exercise and Health Sciences of Ministry of Education, Shanghai University of Sport, Shanghai 200438, China; 18873286059@163.com (L.D.); 18049922807@163.com (Y.Y.); chyang9610@163.com (C.Y.); zhangxini1129@163.com (X.Z.); 2 Department of Mechanical Engineering, Northern Arizona University, Flagstaff, AZ 86011, USA; ying22107@gmail.com; 3 School of Fashion, Beijing Institute of Fashion Technology, Beijing 100029, China

**Keywords:** compression garment, joint mechanics, soft tissue vibrations, muscle activities, drop jump

## Abstract

Objectives: To explore the effects of wearing compression garments on joint mechanics, soft tissue vibration and muscle activities during drop jumps. Methods: Twelve healthy male athletes were recruited to execute drop jumps from heights of 30, 45 and 60 cm whilst wearing compression shorts (CS) and control shorts (CON). Sagittal plane kinematics, ground reaction forces, accelerations of the quadriceps femoris (QF), hamstrings (HM) and shoe heel-cup, and electromyography images of the rectus femoris (RF) and biceps femoris (BF) were collected. Results: Compared with wearing CON, wearing CS significantly reduced the QF peak acceleration at 45 and 60 cm and the HM peak acceleration at 30 cm. Wearing CS significantly increased the damping coefficient for QF and HM at 60 cm compared with wearing CON. Moreover, the peak transmissibility when wearing CS was significantly lower than that when wearing CON for all soft tissue compartments and heights, except for QF at 30 cm. Wearing CS reduced the RF activity during the pre-, post-, and eccentric activations for all heights and concentric activations at 45 cm; it also reduced the BF activity during post- and eccentric activations at 30 and 60 cm, respectively. The hip and knee joint moments and power or jump height were unaffected by the garment type. Conclusion: Applying external compression can reduce soft tissue vibrations without compromising neuromuscular performance during strenuous physical activities that involve exposure to impact-induced vibrations.

## 1. Introduction

The majority of physical activities, including running and jumping, cause collisions between the ground and the human body. Transient shocks and local vibrations are generated, which are either absorbed or transmitted through soft tissues [1,2]. These vibration characteristics, including amplitude and frequency, are tissue-dependent [3,4], and the corresponding muscles are activated in response to the soft tissue vibrations [5].

From a mechanical vibration view, when the frequencies of the impact force and soft tissues are close, resonant oscillations with maximal amplitudes are expected to occur [6]. Nigg et al. [5] proposed a muscle-tuning paradigm to describe muscle responses to resonances to avoid potential microdamage. Briefly, the activation level of affected muscles increases [7,8], and this increment alters the soft tissues’ natural frequencies that help minimize the amplitude of soft tissue vibration and avoid resonant oscillations [8].

However, the additional muscle activation used for damping the soft tissue vibration consumes much energy during a given motor task [7]. In highly controlled situations, an increased O_2_ uptake, which is largely due to extra muscle activation, has been observed in relation to whole-body vibration exercises [9]. This additional amount of energy is estimated to be a small percentage of the total energy used to perform a physical activity [10,11]. However, the influence of this extra energy cannot be considered trivial, especially in prolonged competitions. Several studies have been conducted on the energy consumption when exposed to externally induced vibrations [9,12]. To the best of our knowledge, limited efforts have been exerted on the muscular activation needed to damp soft tissue oscillations in minimally manipulated and strenuous tasks, such as drop jumps (DJs).

As a typical stretch-shortening cycle exercise, DJs are an ‘active landing’ with anticipatory muscle activation confronted with strenuous landing impacts after contact [6]. Immediately after foot contact, muscles are continuously activated to jump up and simultaneously mitigate soft tissue vibration. Separating vibration-induced muscle activity is difficult. As an alternative, muscular reactions can be measured in a situation where the vibrations are extrinsically modified whilst maintaining all other conditions constant. Notably, the external pressures applied by compression garments were shown to dampen soft tissue vibrations during exercise [13,14]. Recently, Broatch et al. [7] reported that wearing compressive garments significantly decreases the medial/lateral and longitudinal displacements of quadricep oscillation following impact. Furthermore, in a reliable study that monitored soft tissue motions with different levels of compression, a significant reduction was observed in the maximal alternation of the distance between markers of none, medium, and high compression during landings [15]. Therefore, compression shorts (CS) have the potential to reduce soft tissue vibrations and satisfy this requirement during jumping and landing tasks. However, only a few studies have reported the effects of CS on vibration-related muscle activation and biomechanical performance during DJs, which further hinders our understanding of the potential mechanisms underlying CG effects on soft tissue vibrations.

Therefore, the current aim was to explore how soft tissue vibration, joint mechanics and muscle activation change in response to compression during DJs. We hypothesized that wearing CS could reduce soft tissue vibration and vibration-induced muscle activity, without compromising lower extremity kinematics, kinetics or jump performance.

## 2. Methods

Twelve male collegiate basketball players (height: 178.3 ± 2.5 cm, body weight: 70.1 ± 4.6 kg, age: 23.7 ± 2.7 years, thigh girth: 55.55 ± 0.81 cm) were recruited. All participants had 5–6 years of experience in playing basketball and had not had any lower extremity musculoskeletal injury within 6 months. Strenuous exercise was not allowed for 24 h prior to the experiment. A sample size of 12 was sufficient, as illustrated in our previous study. A two-tailed *t*-test was performed (G*Power 3.1) to ensure that a sample size of 12 was enough to avoid type II error for all parameters in this study (*p* = 80% at α = 0.05) [16,17]. Informed consent forms approved by the Ethics Committee of Shanghai University of Sport were signed by all participants.

**Figure 1:**
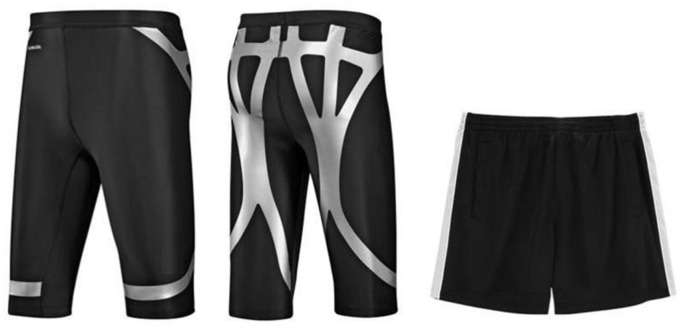
Compression shorts (CS) and control shorts (CON).

According to the manufacturer, the CS was composed of 25% elastane and 75% nylon (Adidas Inc., Herzogenaurach, Germany), and covered the region from the waist to the knees. The pressure applied to the thigh increases gradually from the bottom (16 mmHg) to the top of the quadriceps (24 mmHg), as reported by the manufacturer (Figure 1). The control shorts (CON) were a generic pair of running shorts made of cotton with no tight-fitting feature.

**Figure 2:**
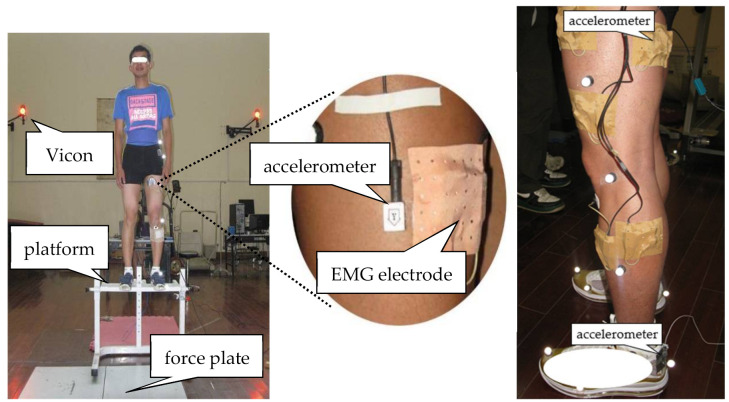
Experimental setup and placements of the accelerometer and electromyography (EMG) electrodes.

Before the formal testing, participants performed 10 min running at 8 km/h and a 5 min static stretch as a warm-up. A successful DJ maneuver was initiated by stepping with both legs off the platform and landing with each foot on two separate force plates with clean footfalls and a good balance; then, a jump with maximum effort was executed after contact (Figure 2) [18]. The participants were required to practice until they felt comfortable performing the task. Before and between trials, there was enough time for participants to rest in case of fatigue. During data collection, each participant attempted to DJ multiple times until three successful trials were completed for each of the three landing heights: 30 (DJ30), 45 (DJ45) and 60 cm (DJ60). The testing order (2 compression × 3 heights) was randomized.

Sagittal plane kinematic data of the dominant leg, defined by the preferred kicking leg [19], were acquired by using an eight-camera infrared 3D motion capture system (Vicon MX, Oxford Metrics, UK) at a sampling rate of 120 Hz with a plug-in gait marker set [17]. The hip, knee, and ankle were defined by twenty-eight retroreflective markers attached to the lower body. Two 90 cm × 60 cm force plates (9287B, Kistler Corporation, Winterthur, Switzerland) laid flat on the surrounding floor were used at 1200 Hz to capture the ground reaction force (GRF). Kinematic data and the GRF were simultaneously collected with the Vicon system.

The quadriceps femoris (QF) and hamstring (HM) vibrations were obtained with two biaxial accelerometers weighing 4 g (Biovision Corp., Wehrheim, Germany) attached to the rectus femoris (RF) and biceps femoris (BF). The measurement range was ±20 g. The first and second accelerometers (Biovision Corp., Wehrheim, Germany) were positioned as shown in Figure 2 by referring to the study of Boyer et al. [20]. The third accelerometer with a measurement range of ±50 g (Biovision Corp., Wehrheim, Germany) was fixed to the heel cup in order to define the impact force (input signal). EMG electrodes were positioned according to the suggestions of SENIAM (Surface EMG for Non-Invasive Assessment of Muscle). EMG signals of BF and RF were collected by using bipolar surface electrodes and the Biovision system (Biovision, Wehrheim, Germany). EMG and acceleration data were collected simultaneously at 1200 Hz. The acquisition system (8.0, DATALOG GmbH, Mönchengladbach, Germany) and Vicon systems were synchronized by using an external trigger.

Sagittal plane kinematic data were filtered through a fourth-order, zero-lag, low-pass Butterworth filter with a cut-off frequency of 7 Hz [21] (Visual 3D v. 5.01.11, C-Motion Inc., Germantown, MD, USA). The hip and knee kinematic parameters included the joint flexion angle and velocity at initial contact (*θ*_cont_ and *ω*_cont_), maximum joint flexion angle (*θ*_max-flx_), and velocity (*ω*_max-flx_), maximum joint extension angle (*θ*_max-ext_) and velocity (*ω*_max-ext_) after foot contact, and the range of motion (*θ*_RoM_) of the joints. The landing phase was defined from ground contact after dropping off the elevated platform to the instant that the toe lifted off the ground. Jump height was calculated by the equation of *v*_0_^2^/2 g (where *v*_0_ is the vertical take-off velocity) [22], reflecting jumping performance.

Sagittal plane hip and knee kinetics included peak joint moments (*M*_max_) and peak joint power (*P*_max_). All kinetic parameters were normalized with respect to body mass.

**Figure 3:**
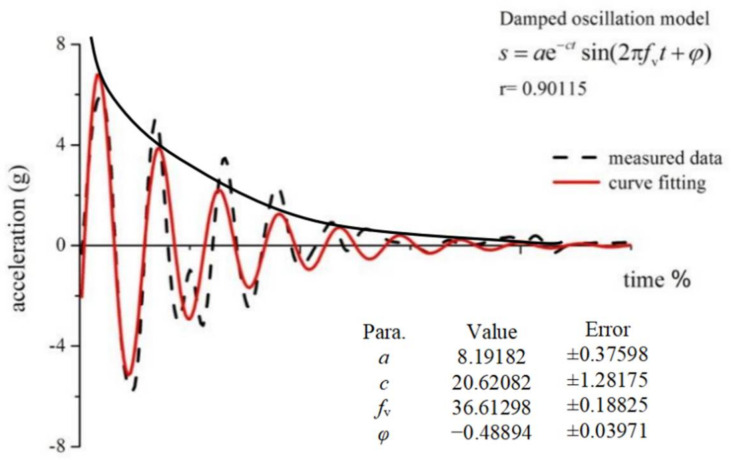
Comparison of the acceleration data of the quadriceps femoris. The solid line denotes the fitted data, and the dashed line denotes the measured data (damped oscillation model: *s* is the measured signal, *a* is the vibration amplitude, *c* is the damping coefficient, *f*_v_ is the dominant frequency, *φ* is the phase coefficient, and *r* is the correlation coefficient).

The peak soft tissue acceleration (*a*_peak_), dominant frequency (*f*_v_), and the damping coefficient (*c*) were used as the main parameters characterizing the vibration (Figure 3) [5,23]. The latter two parameters were determined by the equation below:

$$s=ae^{-ct}\sin(2\pi f_{v}t+\varphi )$$

where *s* is the measured signal, *a* is the vibration amplitude, *c* is the damping coefficient, *f*_v_ is the dominant frequency (damped), and *φ* is the phase coefficient.

The transmissibility (*H*) was used with a modified computing method from a previous study [24], reflecting changes in the vibration characteristics of soft tissues. In brief, the input signal frequency was accessed by the heel cup accelerometer data after fast Fourier transform. The *H*, the ratio of the shoe to the soft tissue compartment acceleration, was calculated by the auto and cross power spectra [6]. The peak transmissibility (*H*_max_) occurred at the resonance frequency, and it represented the resonant oscillation magnitude [25]. Further details about the algorithm of the transfer function can be viewed in our previous work [6].

**Figure 4:**
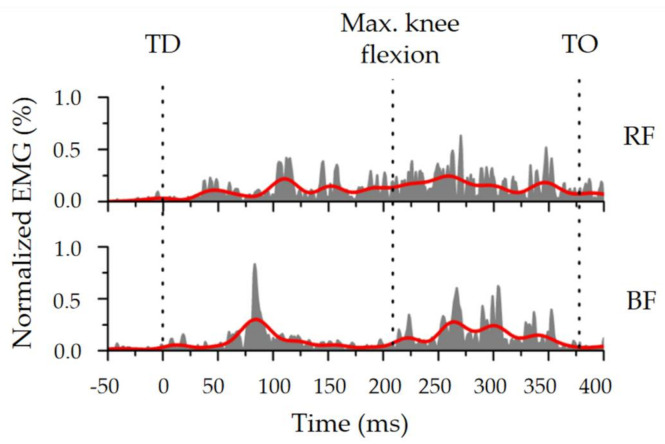
Diagram of the normalized EMG–time curve. RF is rectus femoris, BF is biceps femoris, TD is touchdown, and TO is toe-off.

The EMG data were analyzed using DASYLab software. The raw signals were band-pass-filtered between 10 and 400 Hz to remove the movement artifacts, and then full-wave-rectified [26]. None of the samples were excluded due to the movement artefacts in this study. The EMG amplitudes were normalized as a percentage of the highest value recorded during the 18 DJ trials (Figure 4) [27]. The root mean square of muscle activity (EMG_RMS_) was calculated as

$$EMG_{RMS}=\sqrt{\frac{1}{T}\int_{t}^{t+T}EMG^{2}\left(t\right)\cdot dt}$$

where *t* is the onset of the signal, and *T* is the time interval of each phase.

The four phases were: the pre-activation phase (50 ms before ground contact), post-activation phase (50 ms after touchdown), eccentric phase (from touchdown to the maximum knee flexion) and concentric phase (from the maximum knee flexion to take-off).

A two-way (compression × heights) repeated measures ANOVA was conducted to examine the compression and landing height effects on the joint kinematics and kinetics, soft tissue vibrations, and muscle activities. Tukey’s post hoc analysis was applied if significance was observed (17.0, SPSS Inc., Chicago, IL, USA). The significance level was set as 0.05.

## 3. Results

A significant compression effect was observed amongst *θ*_cont_, *θ*_max_, and *ω*_max_ for the hip joint (Table 1). Specifically, wearing CS significantly increased the hip *θ*_cont_ and *θ*_max-ext_ after foot contact compared with wearing CON for all heights, except for *θ*_max-flx_ at 30 cm (Table 1). Meanwhile, the *θ*_RoM_ values of the hip, knee joint kinematics, and jump height of CS and CON were similar for all heights. Meanwhile, no compression × height interaction, compression effect or height effect was observed for any kinetic parameter (Table 1).

**Figure 5:**
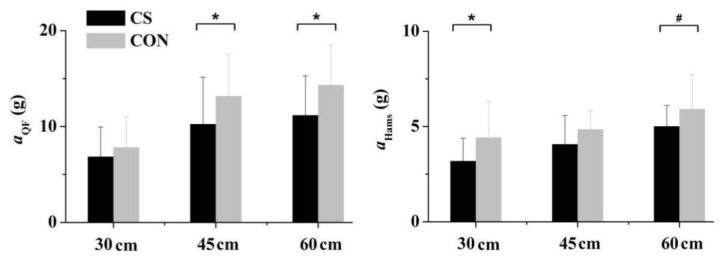
Compression effects on the maximum amplitude (*a*) of quadriceps femoris (QF) and hamstrings (Hams) at different drop heights of landings. * Significantly different from control shorts (CON) at the same landing height with *p* < 0.05; ^#^ different from CON at the same landing height with *p* < 0.1.

There was a significant interaction between compression and landing height for *a*_peak_. Compared with wearing CON, wearing CS significantly reduced the QF *a*_peak_ at 45 and 60 cm, and the HM *a*_peak_ at 30 cm (Figure 5).

**Figure 6:**
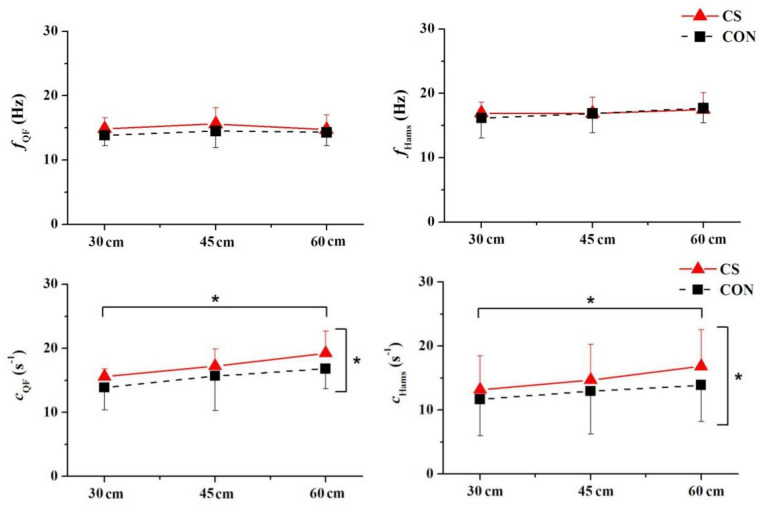
Compression effects on the vibration frequency (*f*) and damping coefficient (*c*) of quadriceps femoris (QF) and hamstrings (Hams) during landings. Upper-pointing brackets (*) indicate a significant difference between 30 cm landing height and 60 cm landing height (*p* < 0.05). Right-pointing brackets (*) indicate a significant difference at 60 cm landing height (*p* < 0.05).

For the dominant frequency, there were no significant differences in the compression conditions for thigh muscles (Figure 6). Wearing CS significantly increased the damping coefficient (*c*) for QF and HM at DJ60 compared with wearing CON. Meanwhile, increasing the landing height from 30 cm to 60 cm significantly increased *c* for QF and HM.

**Figure 7:**
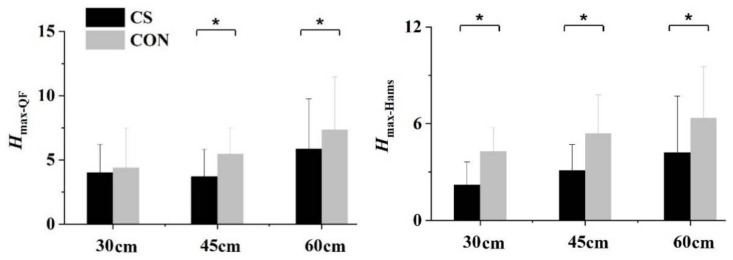
Compression effects on the peak transmissibility (*H*_max_) of quadriceps femoris (QF) and hamstrings (Hams) during landings. Upper-pointing brackets (*) indicate a significant difference at the certain height (*p* < 0.05).

We observed a significant compression–height interaction for *H*_max_. The *H*_max_ values of QF and HM were significantly lower in CS than in CON for all heights, except for the QF at 30 cm (Figure 7).

**Figure 8:**
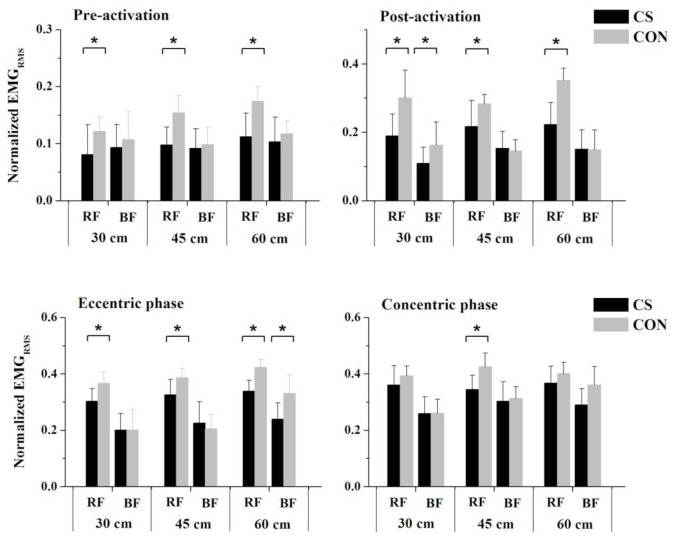
Compression effects on the EMG amplitude of rectus femoris (RF) and biceps femoris (BF) during landings. Upper-pointing brackets (*) indicate a significant difference at the certain height (*p* < 0.05).

Compared with wearing CON, wearing CS significantly reduced the EMG_RMS_ of RF at all phases for all heights, except for the concentric phase when landing from 30 or 60 cm (10 of the 12 parameters), and reduced the EMG_RMS_ of BF in the post-activation and eccentric activation phases when landing from 30 and 60 cm, respectively (Figure 8).

## 4. Discussion

This study evaluated the compression effects on joint mechanics, soft tissue vibrations, and muscular activations during an explosive task (i.e., DJ). The results supported our hypothesis that CS significantly reduces soft tissue vibrations and decreases vibration-related muscle activity during DJ without affecting joint kinetics or jump height.

Wearing CS during DJ did not change the joint mechanics, which supports the findings of other studies. Previous research has indicated that the level of compression does not affect force or power generation in various activities [14,28,29,30,31]. For example, impulse and force development were similar, regardless of the garment type during vertical jumping [32], and the lower body kinetics were identical with and without wearing compression garments during DJs [33]. These findings and the findings of the present study confirm that constrained soft tissue movement from compression does not limit muscle force production or joint torque generation, especially in explosive sports. Moreover, there were no statistical differences in jump height between CS and CON, which were in accordance with the results from a previous study [34]. One possible explanation was that fewer muscle units were recruited due to energy-saving from reduced muscle activation and vibration [28].

The present data suggest that CS effectively attenuated soft tissue vibration by reducing the amplitude of QF and HM vibration. These observations are consistent with the results of previous findings using maximum vertical jump tests, which revealed that relative to wearing CON, wearing CS reduces thigh muscle vibration by 45% and 52% in anterior–posterior and vertical directions, respectively [13]. In previous landing tests, researchers attached markers to the skin to identify soft tissue displacement, and discovered that wearing compression garments with medium to high compactness significantly reduces soft tissue movement compared with wearing garments with no compactness [15]. Collectively, CS work by directly constraining the surface movement of soft tissue, which transmits to the deep layers and reduces the vibration of the entire soft tissue.

Damping coefficient and transmissibility results further elucidate the mechanism underlying the compression effect. The damping coefficients for RF and BF muscles were significantly higher when wearing CS compared with wearing CON during DJ60, supporting previous findings, wherein the damping coefficient for the lower body increased by 8.0 ± 2.1% during ground impact when wearing CS compared to the coefficient for loose-fitting shorts [35]. The peak transmissibility values of QF and HM were significantly lower when wearing CS than when wearing CON at almost all drop heights, indicating a smaller magnitude of resonant oscillation for the former. These findings suggest that applying compression alters damping characteristics rather than changing the natural frequency [2]. Overall, CS provided mechanical forces to the soft tissue surface, which limited the displacement of soft tissue movement. From the perspective of injury prevention, this dampening effect from compression can substantially diminish the impact force from the ground.

This study provides the first evidence that compression can reduce vibration-related muscle activity during strenuous DJ tasks. Compared with wearing CON, wearing CS reduced the EMG_RMS_ of the knee extensors during the pre-, post-, and eccentric activation phases of landing from all heights. Increased muscle pre-activation before impact, considered as ‘muscle tuning’, can reduce soft tissue vibrations [5]. Data show that compression garments provide a similar function as muscle tuning by replacing some of the muscle work, thereby potentially saving energy. In a treadmill running test, runners exerted 1.7% less energy when wearing CS than when wearing regular shorts [36]. Although we did not collect O_2_ uptake, existing data suggest that compression apparel works as a mechanical intervention that can reduce muscle activity and energy expenditure in sports and can slow down fatigue accumulation in prolonged locomotion. Reduced muscle activity has been associated with optimized neurotransmission and sarcomere mechanics at the molecular level [7]. A recent study demonstrated that when completing active movements, the neuromusculoskeletal system regulates certain muscles by increasing their pre- and post-activation phases [3,37]. A reduced muscle activity implies high neuromuscular efficiency during the landing phase of DJ [7,38]. 

This study, however, still has limitations. Firstly, we did not collect energy expenditure data to support the reduced muscle activity. However, we suspect that the metabolic change is negligible, considering that we tested one-time explosive movements. The effect of compression on metabolic data in endurance sports could be further considered. Moreover, the present testing shorts only provided one level of compression for the upper leg. The results should be interpreted with caution when they are applied to CS with different specifications or those that wrap the entire lower extremity. Finally, the acceleration of body segments was not separated from the acceleration of soft tissue vibration.

## 5. Conclusions

The compression shorts reduced the vibration and muscle activation of the quadriceps femoris and hamstrings during drop jumps, but did not influence the hip and knee joint mechanics (joint moment and power) or jump height. These findings provide preliminary evidence that applying external compression can reduce soft tissue vibration without compromising neuromuscular performance. External compression can be further implemented as a mechanical intervention to reduce energy consumption and potentially enhance sports performance during strenuous activities that involve exposure to impact-induced oscillations.

## Figures and Tables

**Table 1:** Comparison of joint kinematics and jump height (mean ± *SD*) of participants wearing CS and CON during DJs at three landing heights.

Drop Height	Shorts Group	Hip Joint							Knee Joint							Jump Height (cm)
		*θ*_cont_ (°)	*θ*_max-flx_ (°)	*θ*_max-ext_ (°)	*θ*_RoM_ (°)	*ω*_cont_ (°/s)	*ω*_max-flx_ (°/s)	*ω*_max-ext_ (°/s)	*θ*_cont_ (°)	*θ*_max-flx_ (°)	*θ*_max-ext_ (°)	*θ*_RoM_ (°)	*ω*_cont_ (°/s)	*ω*_max-flx_ (°/s)	*ω*_max-ext_ (°/s)	
DJ30	CS	37.4 * ±9.9	68.6 ±7.9	13.5 * ±6.8	55.0 ±5.0	180.2 ±55.2	281.8 ±81.0	380.0 ±595	156.2 ±7.7	83.5 ±13.5	3.6 ±4.2	79.9 ±12.7	346.8 ±59.3	559.1 ±55.4	756.5 ±87.2	36.8 ±5.4
	CON	43.8 ±11.9	73.9 ±8.7	18.0 ±5.9	55.8 ±7.7	180.5 ±66.5	266.4 ±89.1	405.2 ±56.3	155.3 ±6.8	80.1 ±10.7	4.6 ±4.9	75.4 ±8.5	330.2 ±60.2	533.0 ±76.0	744.9 ±98.3	37.2 ±4.6
DJ45	CS	33.2 * ±9.6	68.0 * ±6.0	10.6 * ±8.6	57.3 ±7.0	173.0 ±39.4	301.5 ±86.9	382.2 * ±57.5	155.2 ±6.3	87.0 ±13.8	4.1 ±5.0	82.8 ±12.6	400.4 ±63.7	590.7 ±52.9	765.3 ±93.2	37.8 ±7.2
	CON	42.0 ±14.2	76.9 ±7.5	16.8 ±5.5	60.0 ±8.8	187.3 ±58.0	314.6 ±68.8	412.8 ±43.8	155.1 ±4.4	86.0 ±11.5	5.8 ±3.0	80.1 ±9.1	373.0 ±58.3	587.6 ±66.6	761.7 ±73.1	38.7 ±5.4
DJ60	CS	33.7 * ±8.9	71.9 * ±8.0	13.5 * ±6.3	58.3 ±6.8	177.3 ±54.7	308.8 * ±94.9	393.6 * ±53.6	153.6 ±5.5	92.2 ±16.3	4.6 ±4.6	87.5 ±14.6	397.7 ±69.7	593.6 ±64.8	782.3 ±98.2	38.9 ±7.9
	CON	40.4 ±9.6	78.7 ±8.8	16.7 ±6.0	62.0 ±5.2	182.8 ±55.4	372.9 ±85.3	424.5 ±48.4	154.6 ±5.1	87.8 ±13.8	3.5 ±3.4	84.2 ±12.7	396.5 ±63.0	597.4 ±93.7	776.3 ±79.8	39.2 ±5.7

* Significantly different from control shorts (CON) at the same landing height with *p* < 0.05.

## Data Availability

The data presented in this study are available on request from the corresponding author. The data are not publicly available due to restrictions e.g., privacy or ethical.
